# Association of Vitamin D and Incident Statin Induced Myalgia—A Retrospective Cohort Study

**DOI:** 10.1371/journal.pone.0088877

**Published:** 2014-02-19

**Authors:** Ghanshyam Palamaner Subash Shantha, Julio Ramos, Linda Thomas-Hemak, Samir Bipin Pancholy

**Affiliations:** 1 Department of Internal Medicine, The Wright Center for Graduate Medical Education, Scranton, Pennsylvania, United States of America; 2 Department of Cardiovascular Medicine, The Wright Center for Graduate Medical Education, Scranton, Pennsylvania, United States of America; University of Milan, Italy

## Abstract

**Background and Objectives:**

Evidence is conflicting with regards to the role of vitamin D in statin induced myalgia (SIM). Studies so far have assessed cross-sectional association and were limited by study sample selected predominantly from cardiology clinics. In this retrospective cohort study we assessed the association between vitamin D and SIM and attempted to establish a serum vitamin D cutoff to identify patients at risk for developing SIM.

**Methods:**

Medical charts of 5526 consecutive patients from a primary care practice in Scranton, Pennsylvania from 2005–2012 were reviewed. Vitamin D level (25-hydroxy cholecalciferol) at statin initiation was considered “Exposure level”. Vitamin D levels were categorized into quartiles (≤ 10, 11–20, 21–30, >30 ng/ml). SIM was identified by patient report.

**Results:**

1160 out of 5526 patients were treated with statins. The mean age was 55.9 years. 276 (24%) developed SIM. Unadjusted 7-yr cumulative incidences of SIM for quartiles 1–4 of vitamin D were 32.3, 21.5, 18.3 and 14.6% respectively. The lowest quartile of vitamin D was independently associated with 1.21 times the hazard of the fourth quartile for developing SIM (95% CI: 1.09, 1.33; P-trend  = 0.001). Vitamin D cut-off ≤15 ng/ml, showed a positive predictive value, negative predictive value, likelihood ratio (LR) + and LR- of 81, 90, 5.1 and 0.1, respectively for predicting SIM.

**Conclusions:**

Low vitamin D level at statin initiation is associated with SIM, levels ≤15 ng/ml have a high predictive accuracy for SIM. Randomized controlled trials are needed to validate our results.

## Introduction

Statins are effective therapy for primary prevention of cardiovascular events in high risk populations [1], [2]. In patients with cardiovascular disease, statins reduce mortality, morbidity, recurrent cardiovascular events, atrial fibrillation and stroke [1], [2], [3], [4], [5]. Observational studies have reported pleiotropic benefits of statins including reduction in sepsis, pneumonia related mortality [6] and cancer risk [7], though randomized controlled trials have failed to validate these findings.

In spite of proven benefit and the established increased risk of cardiovascular events in patients with coronary artery disease who discontinue statin therapy [8], [9], adherence to statin therapy remains poor and is estimated to be <50% in various patient populations [10], [11], [12], [13]. Among the factors considered, statin induced myalgia (SIM) is reported to the predominant factor associated with poor statin compliance [14], [15]. In a recent study involving multiple primary care practices in Boston, 27% of Statin users reported SIM [16].

Vitamin D has been associated with the development of SIM [17], [18], [19], [20]. However, evidence is conflicting with regards to the cross-sectional association of vitamin D and SIM [17], [18], [19], [20] and studies assessing prospective association between vitamin D and SIM are lacking.

In this retrospective cohort study, involving a large population of unselected patients from a primary care practice in rural Pennsylvania, we have explored the prospective association of vitamin D and SIM and attempted to identify a serum vitamin D cut-off with significant predictive accuracy to identify patients at risk for SIM.

## Materials and Methods

### Study setting and design

This retrospective cohort study was conducted in a primary care outpatient clinic located in Scranton, Pennsylvania. Institutional review board approval was obtained from the Wright Center for Graduate Medical Education institutional review board for conducting the study. Informed consent was not obtained from the participants, as the data was analyzed anonymously per institutional review board guidelines. Institutional review board waived the need for written informed consent from the participants.

Case records of 6946 consecutive adult patients who attended the primary care clinic during the period March 2005 to November 2012 were reviewed. All patients with a minimum of 2 recorded follow-up visits and complete data for the study variables of interest were included in the study. Patient with ≥ stage 3 chronic kidney disease (CKD), decompensated liver disease, decompensated cardiac failure, diagnosed malignancy, and advanced dementia were excluded from the study.

### Baseline data collection

Demographic data (age, gender, race/ethnicity), comorbidities [smoking, diabetes, hypertension, coronary artery disease, rheumatological diagnosis (osteoarthritis, rheumatoid arthritis, fibromyalgia and chronic pain syndrome), psychiatric diagnosis (depression, anxiety, bipolar and post-traumatic stress disorder)], medication history (anti-diabetic medications, anti-hypertensive medications, and lipid-lowering drugs), clinical parameters [height, weight, systolic blood pressure (SBP) and diastolic blood pressure (DBP)], and laboratory parameters [fasting glucose, HbA1c, total cholesterol, low-density lipoprotein cholesterol (LDL), high-density lipoprotein cholesterol (HDL), triglycerides] were recorded. BMI was calculated using standard measures [21].

### Exposure measurement

Serum 25(OH) vitamin D levels, the exposure variable of interest, were assayed using Liaison assay (DiaSorin Inc, Stillwater, MN), a direct enzyme immunoassay method. This assay has a correlation of 0.9 with the gold standard Immuno Radio Assay (IRA), with intra-assay coefficient of variation <8% and an inter-assay coefficient of variation of <10%. 70% of vitamin D samples were analyzed in a lab that was associated with our clinic and the tests for reliability of the vitamin D assay is as mentioned above. The remaining 30% samples were assessed in various labs around Scranton and the measures of reliability for these labs could not be obtained.

### Cohort description

Study participants entered the cohort 1) at the time of enrollment into the clinic if they were already receiving statins and did not report myalgia at enrollment and 2) when they were initiated on statins during follow-up, if they were not on statins at the time of enrollment into the clinic. Participants exited the cohort 1) when they reported statin induced myalgia (SIM), defined as patient reported symptom of muscle ache in the absence of other etiology anytime while on treatment with statins, and 2) when they were administratively censored at study conclusion. Participants who exited due to SIM re-entered the cohort if restarted on any type of statin during follow-up. Vitamin D was assessed as a fixed, time averaged and time variable exposure. For fixed exposure, single value of vitamin D at statin initiation was assessed. This analysis will help us assess temporality in the association between vitamin D levels at statin initiation and incident SIM. For time averaged analysis, all available vitamin D levels from study entry to exit were averaged and the association between this mean vitamin D and incident SIM was assessed. For time variable analysis, participants were considered exposed during times in the follow-up period when they had vitamin D levels ≤30 ng/ml and as unexposed during follow-up periods when vitamin D levels >30 ng/ml. The time averaged exposure analysis gives an assessment of the association of mean vitamin D levels during follow-up and incident SIM. The time variable exposure analysis evaluates the value of the time the patient spent being vitamin D deficient, in predicting incident SIM. Mean number of follow-up visits per patient was 5±3 visits. The decision to stop statins, alter the dose or switch to a different statin was subject to the decision of the attending physician.

### Statistical Analysis

Baseline characteristics were expressed as averages and proportions by quartiles (<10, 11–20, 21–30, >30 ng/ml) of vitamin D levels. Trends across quartiles were evaluated using the median of each quartile as an ordinal variable. Kolmogorov-Smirnov test was applied to assess normality of data distribution. For parametrically distributed variables one way analysis of variance was used to compare across quartiles. For non-parametrically distributed variables Kruskal-Wallis analysis of variance test was used to compare across quartiles.

In the fixed exposure analysis, Cox proportional hazards models were used to examine the association between baseline vitamin D levels (divided into quartiles and as a continuous variable) and risk of SIM using unadjusted and adjusted models [adjusted for age, gender, serum creatinine, hypothyroidism (yes/no) (included subclinical, overt hypothyroidism and euthyroid patients receiving treatment with thyroxine), rheumatologic diagnosis (yes/no) (osteoarthritis needing regular prescription refills of pain pills, diagnosis of rheumatoid arthritis, fibromyalgia, and chronic pain syndrome) and psychiatric diagnosis (depression treated with antidepressants for >2 months at cohort entry, bipolar disorder, anxiety disorder requiring regular prescription refills of anti-anxiety medications and post-traumatic stress disorder)]. Variables namely age, gender, serum creatinine were forced into the adjusted model by virtue of them being possible confounders and then the remaining variables namely hypothyroidism, rheumatologic diagnosis and psychiatric diagnosis were chosen by the process of forward selection of variables due to their significant association with SIM in the unadjusted model. Further, atorvastatin (60%) and simvastatin (29%) were the predominant statins used by our study population. Hence, we tested individually the association of vitamin D and SIM for these 2 statins using the unadjusted and adjusted models by restricting the analysis to the patients using these specific statins.

In the time averaged exposure analysis, average of all available, documented, vitamin D levels during the study period were categorized into quartiles. Then, the association of vitamin D and SIM was assessed (unadjusted and adjusted for variables mentioned above) using logistic regression analysis.

For the time variable exposure analysis, participants were considered exposed during times in the follow-up period when they had vitamin D levels ≤30 ng/ml and as unexposed during follow-up periods when vitamin D levels were >30 ng/ml. Then, in the exposed group follow-up time and incidence of SIM were calculated for vitamin D categories ≤10 ng/ml (corresponds to quartile 1), 11–20 ng/ml (corresponds to quartile 2) and 21–30 ng/ml (corresponds to quartile 3). Similarly, in the unexposed group i.e. vitamin D>30 ng/ml (corresponds to quartile 4) follow-up time and incidence of SIM were calculated. With the time variable exposure analysis, we have attempted to capture an individual participants cumulative exposure (time spent being vitamin D deficient) by their specific vitamin D levels during the times when they were deficient and the duration for which the deficiency existed. Incidence rates of SIM were expressed as number of events/patient weeks of follow-up and were compared across vitamin D quartiles. Logistic regression analysis was performed (unadjusted and adjusted for above mentioned variables) to assess the association of vitamin D (time variable) and SIM.

Further, we performed 2 sensitivity analyses: 1) restricting our sample to those 70% of our study cohort participants who had their vitamin D measured at the single lab associated with our clinic, coding vitamin D as fixed, time averaged and time variable exposure using the unadjusted and adjusted models, 2) restricting our study sample to not include patients who re-entered cohort after re-starting statins, coding vitamin D as fixed, time averaged and time variable exposure using unadjusted and adjusted models.

### Analyses for identifying a 25 (OH) vitamin D cut-off with good predictive accuracy for SIM

Baseline vitamin D cutoffs of ≤5, ≤10, ≤15, ≤20, ≤30 ng/ml were assessed for predictive accuracy for SIM using sensitivity, specificity, positive predictive value, negative predictive value, likelihood ratio of positive test, likelihood ratio of negative test and area under the receiver operating characteristics curve (ROC), using standard methods [22]. Further, we compared the areas under the ROC curves for the various vitamin D cut-offs using the method described by DeLong et. al. [23]. A P-value of <0.05 was considered statistically significant. Statistical analyses were performed using Stata 11.0 statistical software [24].

## Results

From a total of 6946 patient charts reviewed, 1420 patients were excluded for the following reasons; 1056 had missing data for variables of interest (677 had missing values for baseline 25 (OH) vitamin D, 521 had missing 25 (OH) vitamin D at SIM), 106 with ≥ CKD stage 3, 21 with decompensated heart failure, 10 with decompensated liver failure, 5 with advanced dementia, 149 had less than 2 follow-up visits and 73 had missing reason for statin cessation. The remaining 5526 participants formed the study cohort. Compared to the study cohort, the excluded cohort had greater proportion of patients with hypertension, obesity and rheumatologic diagnosis (Table S1 in File S1). In the study cohort, mean serum vitamin D levels were 31.2±14.7 ng/ml (interquartile range 4 to 65 ng/ml) (Table S1 in File S1). Proportions with rheumatologic diagnosis and psychiatric diagnosis increased with decreasing quartiles of vitamin D (Table 1). Over a median follow-up period of 4.2 years (range: 2.3–5.6 years), there were 1160 Statin users (21%) and 276 (24%) of them developed SIM. Of these 276 (24%) who developed SIM, only 9 (0.7%) re-entered the cohort as they were restarted on statins. Statin users who developed SIM had higher proportions of patients with rheumatology diagnosis than statin users who did not develop SIM (Table S2 in File S1). The unadjusted 7-yr cumulative incidences of SIM across quartiles 1–4 of vitamin D concentration were 32.3, 21.5, 18.3, and 14.6% respectively (Figure S1 in File S1).

**Table 1:** Baseline characteristics of study cohort participants by vitamin D quartiles.

Variables	Study cohort	Vitamin D quartiles				P value
		≤10	11–20	21–30	≥31	(trend)
	(n = 5526)	(n = 1404)	(n = 1394)	(n = 1372)	(n = 1384)	
Age (yrs)	61.2 (6.9)	63.4 (8.3)	60.2 (5.3)	63.7 (5.7)	62.1 (8.1)	0.177
Male – n (%)	4237 (61)	1003 (71)	993 (71)	1077 (78)	1164 (84)	0.061
Black – n (%)	764 (11)	201 (14)	175 (13)	189 (14)	199 (14)	0.126
Hypertension- n (%)	1458 (21)	381 (27)	377 (27)	362 (26)	338 (24)	0.152
Diabetes – n (%)	1598 (23)	399 (28)	402 (28)	414 (30)	383 (28)	0.274
Coronary artery disease – n (%)	625 (9)	143 (10)	151 (11)	139 (10)	192 (14)	0.113
Obesity – n (%)	1806 (26)	437 (31)	442 (32)	451 (33)	476 (34)	0.417
Overt hypothyroidism – n (%)	208 (3)	88 (6)	51 (4)	43 (3)	26 (2)	0.031*
SC- hypothyroidism – n (%)	138 (2)	94 (7)	21 (2)	14 (1)	9 (1)	0.021*
Rheumatology disease – n (%)	799 (12)	472 (34)	159 (11)	98 (7)	70 (5)	0.029*
Osteoarthritis – n (%)	607 (9)	367 (26)	123 (9)	61 (4)	56 (4)	<0.001*
Rheumatoid arthritis – n (%)	71 (1)	45 (3)	12 (1)	10 (1)	4 (0.3)	0.073
Fibromyalgia – n (%)	48 (0.7)	17 (1)	5 (0.4)	16 (1)	10 (1)	0.159
Chronic pain syndrome – n (%)	73 (1)	43 (3)	19 (1)	11 (1)	0 (0)	0.045
Current smoking – n (%)	1250 (18)	275 (20)	287 (21)	292 (21)	396 (27)	0.117
Psychiatric diagnosis – n (%)	1319 (19)	639 (46)	277 (20)	208 (15)	195 (14)	0.019
Depression – n (%)	778 (14)	518 (37)	136 (10)	88 (6)	36 (3)	<0.001*
Anxiety – n (%)	430 (8)	100 (7)	105 (8)	86 (6)	139 (10)	0.221
Bipolar – n (%)	101 (2)	18 (1)	34 (2)	31 (2)	18 (1)	0.376
PTSD – n (%)	10 (0.2)	3 (0.2)	2 (0.2)	3 (0.2)	2 (0.1)	0.881
Mean creatinine (mg/dl)	0.97 (0.22)	0.99 (0.27)	0.98 (0.21)	0.98 (0.25)	0.97 (0.22)	0.336
Mean hemoglobin (g/dl)	13.7 (1.3)	13.4 (1.6)	13.8 (1.8)	13.5 (1.9)	13.1 (1.9)	0.219
HbA1C%	5.5 (1.7)	5.4 (1.8)	5.5 (1.5)	5.6 (1.4)	5.5 (1.6)	0.177
Total cholesterol (mmol/l)	5.3±1.2	5.4±1.3	5.3±1.2	5.5±1.4	5.1±1.3	0.264
LDL cholesterol (mmol/l)	4.2±1.0	4.1±0.8	4.3±1.1	4.2±1.1	4.0±0.9	0.511
Triglycerides (mmol/l)	1.8±0.3	1.9±0.3	1.7±0.4	1.8±0.3	1.9±0.4	0.380

PTSD: post-traumatic stress disorder, *represents statistically significant associations

In the fixed exposure analysis, vitamin D measured at statin initiation, when examined as a continuous variable with adjustment for all covariates, every 1 ng/ml decrease in vitamin D levels was associated with 1.22 times the hazard of SIM (model 2, 95% CI: 1.09, 1.36). The lowest quartile of vitamin D had significantly greater hazard of SIM (Hazards ratio: 1.20, 95% C.I: 1.06–1.38) compared to the highest quartile of vitamin D (Table 2). When atorvastatin and simvastatin users were assessed individually, the association between SIM and Vitamin D level remained robust (Table 2).

**Table 2:** Association of vitamin D and SIM.

	Vitamin D quartiles (ng/ml)				P-trend
	≤≤10	11–20	21–30	≥31	
Fixed exposure analysis*					
Unadjusted	1.21 (1.07–1.44)	1.15 (1.03–1.39)	1.08 (1.00–1.22)	Ref = 1	<0.001
Adjusted	1.20 (1.06–1.38)	1.13 (1.03–1.31)	1.09 (0.90–1.24)	Ref = 1	0.011
Sensitivity analysis$ (adjusted model)	1.29 (1.04–1.58)	1.21 (1.08–1.29)	1.06 (1.01–1.15)	Ref = 1	<0.001
Sensitivity analysis& (adjusted model)	1.20 (1.04–1.37)	1.15 (1.03–1.30)	1.09 (1.00–1.23)	Ref = 1	<0.001
Atorvastatin users (adjusted model)	1.36 (1.13–1.59)	1.29 (1.10–1.64)	1.03 (0.92–1.45)	Ref = 1	<0.001
Simvastatin users (adjusted model)	1.44 (1.09–1.82)	1.31 (1.09–1.77)	1.11 (1.01–1.49)	Ref = 1	<0.001
Time averaged exposure analysis#					
Unadjusted	1.39 (1.15–1.49)	1.29 (1.09–1.42)	1.14 (1.05–1.31)	Ref = 1	<0.001
Adjusted	1.26 (1.10–1.41)	1.13 (1.07–1.38)	1.08 (0.99–1.24)	Ref = 1	<0.001
Sensitivity analysis$ (adjusted model)	1.35 (1.12–1.61)	1.28 (1.07–1.33)	1.10 (1.06–1.27)	Ref = 1	<0.001
Sensitivity analysis& (adjusted model)	1.24 (1.11–1.37)	1.14 (1.05–1.36)	1.08 (1.02–1.26)	Ref = 1	<0.001
Time variable exposure analysis#					
Unadjusted	1.47 (1.21–1.76)	1.30 (1.14–1.62)	1.14 (1.07–1. 47)	Ref = 1	<0.001
Adjusted	1.40 (1.27–1.58)	1.29 (1.16–1.68)	1.06 (0.97–1.27)	Ref = 1	<0.001
Sensitivity analysis$ (adjusted model)	1.42 (1.19–1.68)	1.36 (1.11–1.51)	1.15 (1.03–1.37)	Ref = 1	<0.001
Sensitivity analysis& (adjusted model)	1.41 (1.25–1.60)	1.27 (1.15–1.61)	1.05 (0.96–1.29)	Ref = 1	<0.001

*: estimates are Hazards ratio (95% C.I), #: estimates are odds ratios (95% C.I), $: sample restricted to those patients whose vitamin D levels were measured at the single lab associated with our clinic. &: study sample after excluding the 9 patients who re-entered cohort after restarting statins.

When vitamin D was treated as time averaged variable, the unadjusted cumulative incidence of SIM across quartiles 1–4 of vitamin D concentration were 30.1, 24.5, 17.5 and 12.9% respectively. After adjustment, association of vitamin D and SIM was robust (quartile 1 compared to quartile 4) (odds ratio: 1.26, 95% CI: 1.10–1.41) (Table 2).

In the time variable analysis, cumulative incidence of SIM over 7 years follow-up was 627/100 weeks follow-up. The unadjusted incidence of SIM across quartiles 1–4 of vitamin D concentration were respectively 401/100 weeks follow-up, 107/100 weeks follow-up, 70/100 weeks follow-up and 49/100 weeks of follow-up. After adjustment, association of vitamin D and SIM was strong and independent (odds ratio: 1.40, 95% C.I: 1.27–1.58).

When the analysis was restricted to 3868 (70%) patients from the study cohort who had their vitamin D measured in the single lab that was associated with our clinic (sensitivity analysis), there were 890 (23%) statin users and 231 (26%) developed SIM. There was an independent association between Vitamin D and SIM in the fixed exposure, time averaged and time variable exposure analysis (Table 2). In the second sensitivity analysis, after excluding the 9 patients (0.7%) who re-entered cohort after restarting statins, the association between vitamin D and SIM remained robust in the fixed exposure, time averaged exposure and time variable exposure analyses (Table 2).

Among the various vitamin D cut-offs assessed, values ≤15 ng/ml, showed a sensitivity of 89%, specificity of 83%, positive predictive value of 81%, negative predictive value of 90%, area under the curve (AUC) of 79%, likelihood ratio (LR) + and LR- of 5.1 and 0.1, respectively in predicting SIM (Table 3 and Figure S2 in File S1, Figure S3 in File S1). When the various vitamin D cut-offs were compared, cut-off ≤15 ng/ml had a better predictive accuracy for SIM when compared to cut-off ≤5 ng/ml (P<0.001), ≤10 ng/ml (P = 0.017), ≤20 ng/ml (P = 0.001) and ≤30 ng/ml (P<0.001). When vitamin D was coded as a continuous variable, the AUC for predicting SIM was 89% (Figure S4).

**Table 3:** Predictive accuracy of vitamin D cut-offs.

Parameters	Vitamin D cut-offs (ng/ml)				
	≤5	≤10	≤15	≤20	≤30
Sensitivity	22	66	89	88	92
Specificity	90	90	83	78	77
Positive predictive value	62	85	81	80	73
Negative predictive value	60	76	90	66	58
Area under the curve	51	62	79	65	53
Likelihood ratio +	2.2	6.8	5.1	2.0	1.4
Likelihood ratio −	0.8	0.3	0.1	0.2	0.3
Number developed SIM	59	84	192	211	231
Total statin users	137	288	434	547	716

## Discussion

Our study involving a large sample of unselected patients from a primary care practice in rural Pennsylvania has observed an independent and prospective association between vitamin D and SIM. Further, serum vitamin D level ≤15 ng/ml at statin initiation is observed to have a high accuracy in predicting occurrence of SIM.

Mechanism underlying SIM is unclear and is thought to be due to reduction in muscle mitochondrial levels of coenzyme Q10 (CoQ10) that has a role in muscle energy production, subsequent to inhibition of mevalonate pathway by statins [25]. Further, skeletal muscle levels of plant sterols are increased by nearly 50% in high dose statin users [26], which by inhibiting acetyl-CoA carboxylase, reduces fat synthesis, increases beta-oxidation and results in muscle injury [26]. Individual genetic susceptibility also seems to play a role in SIM [27]. Potential mechanistic link for vitamin D in this process is that statins inhibit CYP3A4, which has 25- hydroxylase activity in-vitro [28]. With vitamin D deficiency, CYP3A4 may potentially get shunted for vitamin D hydroxylation, in an effort to maintain levels of 25 (OH) vitamin D, reducing availability of CYP3A4 for statin metabolism, thereby increasing serum statin levels and subsequent statin toxicity.

Epidemiological data is conflicting with regards to the role of vitamin D in SIM. Cross sectional studies have argued for [17], [18] and against [19], [20] this association. A recent systematic review concluded that evidence is unclear with regards to the association of vitamin D and SIM and suggested the need for further studies examining this association [29]. Establishing the mechanism behind this association or determining causality is beyond the scope of our paper. However, we observed robust association of vitamin D and SIM in the longitudinal analysis, treating vitamin D levels as a fixed exposure variable, time averaged exposure variable and time variable exposure variable. Considering the temporality of events, with vitamin D deficient patients, without myalgia at statin initiation, subsequently developing myalgia after statin therapy adds validity to our hypothesis associating vitamin D with SIM and negates the possibility of reverse causation that prior cross-sectional studies in this topic were criticized for [17]–[20]. Exposure misclassification is possible in our study as there was no uniformity in the frequency of assessment of vitamin D. However, since this misclassification was random, the bias in the effect estimate would have been conservative. In spite of this, we observed a robust association between vitamin D level and SIM. Also, our observational data might have had significant confounding due to psychiatric diagnosis and rheumatologic diagnosis (Table 2). However, association of vitamin D and SIM remained strong even after adjustment for these variables (Table 2), corroborating an independent relationship between vitamin D and SIM.

Our study has shown that serum vitamin D cut-off ≤15 ng/ml has high accuracy in predicting SIM. We note that when a cut-off of a serum level of a laboratory parameter is assessed for its predictive accuracy, the prevalence of the event of interest should be taken into consideration [22]. When the prevalence of the event of interest (SIM) is high (24% in our study), values of sensitivity, specificity, positive predictive value and negative predictive value may be less useful for clinical application, since all of the above-mentioned indicators of test accuracy vary with prevalence. In this context, the indicators of test accuracy which do not significantly vary with prevalence are the most clinically relevant. LR+ and LR- do not vary with prevalence of the disease [22]. An LR+ >1 and an LR− <1 makes a test clinically meaningful, while a LR+ >10 and LR− <0.1 indicates very high accuracy [22]. In our study, among all vitamin D cut-offs assessed, Levels ≤15, with its LR+ of 5.1 and LR− of 0.1, had the highest predictive accuracy. However, from Table 3, we can see that any serum vitamin D level ≤30 ng/ml satisfied the criteria for LR+ >1 and LR−<1, and hence are good predictors for SIM, emphasizing the need to address any level of vitamin D deficiency at statin initiation.

### Limitations

We observed a higher incidence of SIM (24%) compared to prior observational studies [17], [18], [19], [20], [30] that reported SIM (10–15%). Though our sample size was larger and we had a longer follow-up, it is possible that patient and clinic characteristics may have played a role in this observed higher incidence. Retrospective cohort design limited us to the data that could be obtained from chart review. Risk factors for myalgia that were controlled in other studies such as parathyroid status and bone turnover markers were unavailable [31]. Atorvastatin (60%) and Simvastatin (29%) were the predominant statins used by our patients. Hence our findings should not be extrapolated to other statins. Another important limitation is that we could not adequately account for effect of vitamin D supplementation on SIM as there was no consistent documentation of treatment dose, duration, compliance and vitamin D levels after replacement at the time of or after SIM diagnosis.

## Conclusion

Our study shows an association between vitamin D deficiency at statin initiation and subsequent development of SIM. Though this prospective association between vitamin D and incident SIM is applicable to statin users, our study results should not be inferred to support an interaction between vitamin D and statin use on myalgia, as our study was not designed to assess this hypothesis. Our observational data needs validation with prospective randomized trials (RCT) with vitamin D replacement at statin initiation and its effect on the future development of SIM. Such RCTs can validate our identified vitamin D cut-off of ≤15 ng/ml for its predictive accuracy of SIM.

## Supporting Information

File S1 **Figure S1, Cummulative unadjusted incidence of SIM across vitamin D quartiles. Figure S2, Predictive accuracy of vitamin D cut-offs (ng/ml). Figure S3, Area under the curve for vitamin D cut-off of ≤15 ng/ml. Figure S4, Area under the curve when vitamin D was coded as a continuous variable. Table S1, Comparison between study cohort and excluded cohort. Table S2, Comparison between statin users with SIM and statin users without SIM.**
(DOC)
