# Supplementary material for: Association of Vitamin D and Incident Statin Induced Myalgia—A Retrospective Cohort Study
Source: PLoS One. 2014 Feb 19;9(2):e88877. doi: 10.1371/journal.pone.0088877 (PMC3929495; doi:10.1371/journal.pone.0088877)
Supplement: File S1 — Figure S1, Cummulative unadjusted incidence of SIM across vitamin D quartiles. Figure S2, Predictive accuracy of vitamin D cut-offs (ng/ml). Figure S3, Area under the curve for vitamin D cut-off of ≤15 ng/ml. Figure S4, Area under the curve when vitamin D was coded as a continuous variable. Table S1, Comparison between study cohort and excluded cohort. Table S2, Comparison between statin users with SIM and statin users without SIM. (DOC) [file pone.0088877.s001.doc]

**File S1: Supporting information file that combines Table S1, Table S2, Figure S1, Figure S2, Figure S3 and Figure S4**

**Table S**1: Comparison between study cohort and excluded cohort

| Variables | Overall | Study cohort | Excluded cohort | P-value |
| --- | --- | --- | --- | --- |
|  | (n = 6946) | (n = 5526) | (n = 1420) |  |
| Age (yrs) | 61.2 (6.9) | 63.2 (7.7) | 60.5 (8.2) | 0.117 |
| Male – n (%) | 4237 (61) | 3400 (62) | 837 (59) | 0.133 |
| Black – n (%) | 764 (11) | 585 (11) | 179 (13) | 0.261 |
| Hypertension- n (%) | 1458 (21) | 1090 (20) | 368 (26) | 0.042 |
| Diabetes – n (%) | 1598 (23) | 1273 (23) | 325 (22) | 0.610 |
| Coronary artery disease – n (%) | 625 (9) | 497 (8) | 128 (9) | 0.611 |
| Obesity – n (%) | 1806 (26) | 1370 (25) | 436 (30) | 0.037* |
| Overt hypothyroidism – n (%) | 208 (3) | 167 (3) | 41 (3) | 0.793 |
| SC- hypothyroidism – n (%) | 138 (2) | 106 (2) | 22 (2) | 0.794 |
| Rheumatology disease – n (%) | 799 (12) | 563 (10) | 236 (17) | 0.024 |
| Osteoarthritis – n (%) | 607 (9) | 504 (9) | 103 (7) | 0.091 |
| Rheumatoid arthritis – n (%) | 71 (1) | 27 (0.5) | 44 (3) | 0.037* |
| Fibromyalgia – n (%) | 48 (0.7) | 17 (0.3) | 31 (2) | 0.039* |
| Chronic pain syndrome – n (%) | 73 (1) | 15 (0.3) | 58 (4) | 0.024* |
| Psychiatric diagnosis – n (%) | 1319 (19) | 998 (18) | 321 (23) | 0.066 |
| Depression – n (%) | 778 (11) | 612 (11) | 166 (12) | 0.128 |
| Anxiety – n (%) | 451 (7) | 335 (6) | 116 (8) | 0.104 |
| Bipolar – n (%) | 80 (1) | 63 (1) | 17 (1) | 0.114 |
| PTSD – n (%) | 10 (0.2) | 8 (0.1) | 2 (0.1) | 0.288 |
| Current smoking – n (%) | 1250 (18) | 969 (18) | 281 (20) | 0.122 |
| Mean creatinine (mg/dl) | 0.97 (0.22) | 0.96 (0.19) | 1.2 (0.24) | 0.081 |
| Mean hemoglobin (g/dl) | 13.7 (1.3) | 13.9 (1.9) | 13.4 (1.6) | 0.352 |
| HbA1C% | 5.5 (1.7) | 5.4 (1..2) | 5.5 (0.9) | 0.226 |
| Total cholesterol (mmol/l) | 5.3 (1.2) | 5.2 (1.3) | 5.4 (1.7) | 0.103 |
| LDL cholesterol (mmol/l) | 4.2 (1.0) | 4.1 (0.8) | 4.1 (1.3) | 0.226 |
| Triglycerides (mmol/l) | 1.8 (0.3) | 1.7 (0.3) | 1.9 (0.2) | 0.551 |
| Mean vitamin D levels (ng/ml) | 33.1 (10.8) | 31.2 (14.7) | 33.7 (12.6) | 0.773 |

*represent statistically significant association.

Continuous variables in the table repesent mean (standard deviation); SC: subclinical; PTSD: post-traumatic stress disorder.

**Table S2: comparison between statin users with SIM and statin users without SIM**

| Variables | Statin users | Statin users with SIM | Statin users without SIM | P value |
| --- | --- | --- | --- | --- |
|  | (n = 1160) | (n = 276) | (n = 884) |  |
| Age (yrs) | 62.1 (10.9) | 63.5 (10.1) | 61.8 (13.9) | 0.180 |
| Male – n (%) | 719 (62) | 184 (67) | 535 (61) | 0.086 |
| Black – n (%) | 104 (9) | 39 (14) | 65 (7) | 0.060 |
| Hypertension- n (%) | 464 (40) | 110 (40) | 354 (40) | 0.961 |
| Diabetes – n (%) | 394 (34) | 97 (35) | 297 (34) | 0.518 |
| Coronary artery disease – n (%) | 267 (23) | 66 (24) | 201 (23) | 0.443 |
| Obesity – n (%) | 650 (56) | 160 (58) | 490 (56) | 0.152 |
| Overt hypothyroidism – n (%) | 116 (10) | 31 (11) | 85 (10) | 0.337 |
| SC- hypothyroidism – n (%) | 70 (6) | 16 (6) | 54 (6) | 0.896 |
| Rheumatology disease – n (%) | 244 (21) | 114 (41) | 130 (15) | 0.031* |
| Osteoarthritis – n (%) | 177 (15) | 87 (32) | 90 (10) | 0.037* |
| Rheumatoid arthritis – n (%) | 46 (4) | 20 (7) | 26 (3) | 0.042* |
| Fibromyalgia – n (%) | 10 (1) | 3 (1) | 7 (1) | 0.119 |
| Chronic pain syndrome – n (%) | 11 (1) | 4 (1) | 7 (1) | 0.119 |
| Current smoking – n (%) | 313 (27) | 77 (28) | 236 (27) | 0.101 |
| Psychiatric diagnosis – n (%) | 267 (23) | 65 (24) | 202 (23) | 0.417 |
| Depression – n (%) | 129 (11) | 39 (14) | 90 (10) | 0.099 |
| Anxiety – n (%) | 107 (9) | 12 (4) | 95 (11) | 0.060 |
| Bipolar – n (%) | 23 (2) | 11 (4) | 12 (1) | 0.107 |
| PTSD – n (%) | 8 (1) | 3 (1) | 5 (1) | 0.889 |
| Mean creatinine (mg/dl) | 0.92 (0.2) | 0.89 (0.3) | 0.91 (0.3) | 0.113 |
| Mean hemoglobin (g/dl) | 13.6 (1.0) | 13.8 (1.4) | 13.2 (1.6) | 0.420 |
| HbA1C% | 5.4 (0.9) | 5.4 (1.0) | 5.6 (1.3) | 0.255 |
| Total cholesterol (mmol/l) | 5.4 (1.3) | 5.4 (1.4) | 5.3 (1.1) | 0.286 |
| LDL cholesterol (mmol/l) | 4.3 (1.2) | 4.2 (1.0) | 4.3 (0.9) | 0.377 |
| Triglycerides (mmol/l) | 1.8 (0.3) | 1.9 (0.2) | 1.9 (0.3) | 0.462 |
| Mean vitamin D levels (ng/ml) | 31.2 (14.7) | 22.3 ± 7.1 | 33.8 ± 4.5 | 0.039* |

SC: sub-clinical hypothyroidism, PTSD: post-traumatic stress disorder, *represents statistically significant association

**Figure S1: Cummulative unadjusted incidence of SIM across vitamin D quartiles**

Quartile 1: ≤10 ng/ml; Quartile 2: 11 – 20 ng/ml; Quartile 3: 21 – 30 ng/ml, Quartile 4: > 30 ng/ml

**Figure S2: Predictive accuracy of vitamin D cut-offs (ng/ml)**

**Figure S3: Area under the curve for vitamin D cut-off of ≤ 15 ng/ml**

**Figure S4**: **Area under the curve when vitamin D was coded as a continuous variable**
